# Thymol Mitigates Cadmium Stress by Regulating Glutathione Levels and Reactive Oxygen Species Homeostasis in Tobacco Seedlings

**DOI:** 10.3390/molecules21101339

**Published:** 2016-10-14

**Authors:** Xiefeng Ye, Tianxiao Ling, Yanfeng Xue, Cunfa Xu, Wei Zhou, Liangbin Hu, Jian Chen, Zhiqi Shi

**Affiliations:** 1 College of Tobacco Science, Henan Agricultural University, Zhengzhou 450002, China; yexiefeng@163.com (X.Y.); 18837103304@163.com (T.L.); 2 Nanjing Yangzi Modern Agriculture Investment and Development Co. Ltd., Nanjing 211899, China; hnndxyf@163.com; 3 Central Laboratory, Jiangsu Academy of Agricultural Science, Nanjing 210014, China; jaasxucunfa@163.com; 4 Department of Food Science, Henan Institute of Science and Technology, Xinxiang 453003, China; zhouweihistfood@163.com (W.Z.); hulb973@163.com (L.H.); 5 Institute of Food Quality and Safety, Jiangsu Academy of Agricultural Sciences, Nanjing 210014, China; 6 Key Laboratory of Food Quality and Safety of Jiangsu Province-State Key Laboratory Breeding Base, Jiangsu Provincial Department of Agriculture and Forestry, Nanjing 210014, China

**Keywords:** thymol, reactive oxygen species, cell death, glutathione, cadmium, tobacco

## Abstract

Thymol is a famous plant-derived compound that has been widely used in pharmacy due to its antioxidant and antimicrobial properties. However, the modulation of intrinsic plant physiology by thymol remains unclear. It is a significant challenge to confer plant tolerance to Cd (cadmium) stress. In the present study physiological, histochemical, and biochemical methods were applied to investigate thymol-induced Cd tolerance in tobacco (*Nicotiana tabacum*) seedlings. Thymol was able to alleviate Cd-induced growth inhibition of tobacco seedlings in both dose- and time-dependent manners. Both histochemical detection and in-tube assays suggested that thymol treatment blocked Cd-induced over-generation of reactive oxygen species (ROS), lipid peroxidation, and loss of membrane integrity in both leaves and roots. Thymol decreased Cd-induced cell death that was indicated in vivo by propidium iodide (PI) and trypan blue, respectively. Thymol stimulated glutathione (GSH) biosynthesis by upregulating the expression of γ-*glutamylcysteine synthetase 1* (*GSH1*) in Cd-treated seedlings, which may contribute to the alleviation of Cd-induced oxidative injury. In situ fluorescent detection of intracellular Cd^2+^ revealed that thymol significantly decreased free Cd^2+^ in roots, which could be explained by the thymol-stimulated GSH biosynthesis and upregulation of the expression of *phyochelatin synthase 1* (*PCS1*). Taken together, these results suggested that thymol has great potential to trigger plant resistant responses to combat heavy metal toxicity, which may help our understanding of the mechanism for thymol-modulated cell metabolic pathways in response to environmental stimuli.

## 1. Introduction

Large amounts of Cd (cadmium) have been released into the environment by both natural and anthropogenic process, which has been drawing great attention worldwide [1]. In the Cd-contaminated agricultural environment, the ionic cadmium (Cd^2+^) can be readily taken up by plants, leading to phytotoxicity and posing potential risk to human health through the food chain [2]. One of the most important toxic effects of Cd stress on plants is to induce the accumulation of reactive oxygen species (ROS), which further triggers oxidative injury, stress-responsive signaling, and cell death [3]. Among the resistant strategies developed by plants, glutathione (GSH) plays dual functions in combating Cd toxicity. First, GSH acts as an intracellular antioxidant to maintain cellular redox homeostasis by scavenging Cd-induced ROS in plants [4]. Second, GSH is an indispensable precursor for the biosynthesis of phytochelatins (PCs). PCs are composed of small cysteine-rich peptides, which can chelate and compartmentalize Cd to attenuate cytotoxicity induced by free Cd in plant cells [5,6]. Both the GSH-biosynthetic gene *GSH1* (*γ-glutamylcysteine synthetase 1*) and the PCs-biosynthetic gene *PCS1* (*phyochelatin synthase 1*) have been demonstrated to be critical for Cd detoxification in plants [7,8]. Remediation of Cd-contaminated soils is the fundamental method to solve the problem of Cd-induced eco-toxicity, but it is hard to apply extensively due to some disadvantages (such as high cost or being time-consuming) [9]. Recently, exogenous regulation of intrinsic plant physiology has been recommended as a simple alternative to decrease Cd-induced phytotoxicity in Cd-contaminated environment [10,11].

Thymol [5-methyl-2-(1-methylethyl) phenol] is the main constituent of oils obtained from *Thymus* *vulgaris*. Thymol is considered as a natural monoterpene phenol that has been highly appreciated for its clinical relevance and antimicrobial activity [12,13]. It has been reported that thymol shows anti-inflammatory and anti-oxidative properties by triggering immune responses in mammalian cells [14,15,16]. Thymol can be applied as a food preservative to maintain the quality of fruits and vegetables during postharvest storage [17,18]. Based on the evaluation of the U.S. Environmental Protection Agency (EPA) Office of Pesticide Programs, thymol has minimal potential toxicity and poses minimal risk [19]. In addition, the U.S. Food and Drug Administration (FDA) has listed thymol as a Generally Recognized as Safe (GRAS) substance [20]. Therefore, thymol has great potential to be applied as a safe bio-agent in agriculture. Although thymol is able to protect mammalian cells from environmental stimuli [21], whether and how thymol can modulate intrinsic plant physiology is still elusive.

In this study, we investigated the protecting effect of thymol on Cd-induced growth inhibition in tobacco (*Nicotiana tabacum*) seedlings. The role of thymol in the attenuation of ROS accumulation and oxidative injury in Cd-treated seedlings was further confirmed. Finally, the role of thymol-induced endogenous GSH biosynthesis was studied. The possible mechanism of thymol driving these physiological processes, and their significance, are discussed as well.

## 2. Results

### 2.1. Thymol Significantly Alleviated Cd-Induced Growth Inhibition of Tobacco Seedlings

**Figure 1:**
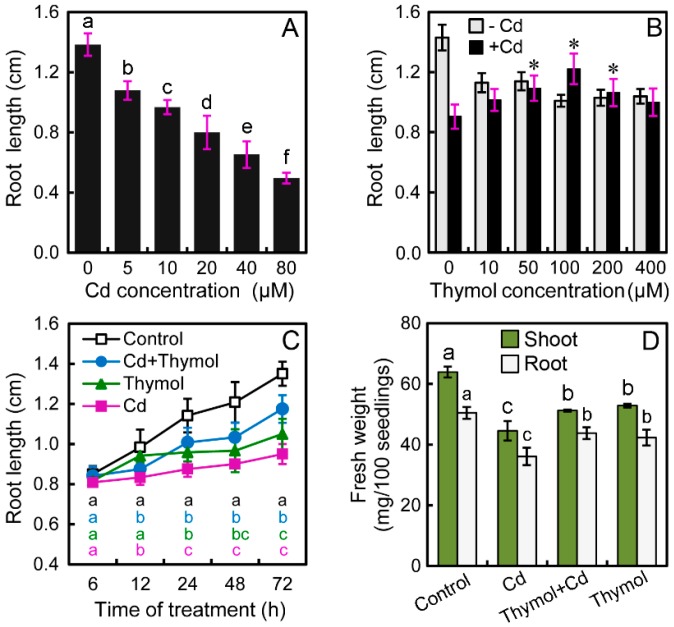
The effect of thymol on the growth of tobacco seedlings under Cd stress. (**A**) The roots of seedlings were treated with CdCl_2_ at 0–80 μM for 72 h for the measurement of root length; (**B**) In the presence of CdCl_2_ at 20 μM, the roots of seedlings were treated with thymol at different concentrations (0–400 μM) for 72 h. Then the root length was measured; (**C**) The roots of seedlings were exposed to 20 μM of CdCl_2_ and 100 μM of thymol simultaneously for 6, 12, 24, 48, and 72 h, respectively, for the measurement of root length; (**D**) The roots of seedlings were treated with water, 20 μM of CdCl_2_, 100 μM of thymol, alone, or their combinations for 72 h. Then the shoots and roots were harvested, respectively, for the quantification of fresh weight. Each value was represented as the mean of three replicates with SD. Different letters in (**B**) and (**D**) indicate that the mean values are significantly different between the treatments (*p* < 0.05, ANOVA, LSD). Different letters in (**C**) indicate that the mean values are significantly different among four treatments at a particular point in time (*p* < 0.05, ANOVA, LSD). The asterisk in (**B**) indicates that the mean value was significantly different between thymol + Cd treatment and Cd treatment alone (*p* < 0.05).

Treatment with CdCl_2_ remarkably inhibited the root growth of tobacco seedlings in a dose-dependent manner. The roots were exposed to CdCl_2_ at 0–80 μM for 72 h. Compared to the control, the root length decreased by 22.05%, 30.04%, 42.21%, 52.85%, and 64.14% at 5, 10, 20, 40, and 80 μM of Cd levels, respectively (Figure 1A). Treatment with Cd at 20 μM was used for further experiments. To understand the effect of thymol on the regulation of root growth under Cd stress, thymol with different concentrations (0–400 μM) were added to the treatment solution. Under normal conditions without Cd stress, thymol treatment inhibited the root growth of seedlings. However, compared to Cd treatment alone, the addition of thymol at 50–200 μM resulted in the significant increase in root length. Thymol at 100 μM showed the greatest effect on the alleviation of Cd-induced inhibition of root elongation (Figure 1B). In a time-course experiment, the Cd-induced decrease in root length was significantly recovered when roots were incubated in the treatment solution containing both Cd and 100 μM of thymol. At the end of the experiment (up to 72 h), the root length under Cd + thymol treatment significantly increased by 35.10% as compared to Cd treatment alone (Figure 1C). In addition, thymol significantly enhanced the fresh weight of both shoots and roots under Cd stress (Figure 1D). Thymol treatment alone showed a slight inhibitory effect on seedling growth (Figure 1B–D), but these results suggested that thymol recovered the growth of tobacco seedlings from Cd stress.

### 2.2. Thymol Blocked Cd-Induced ROS Accumulation in Tobacco Seedlings

**Figure 2:**
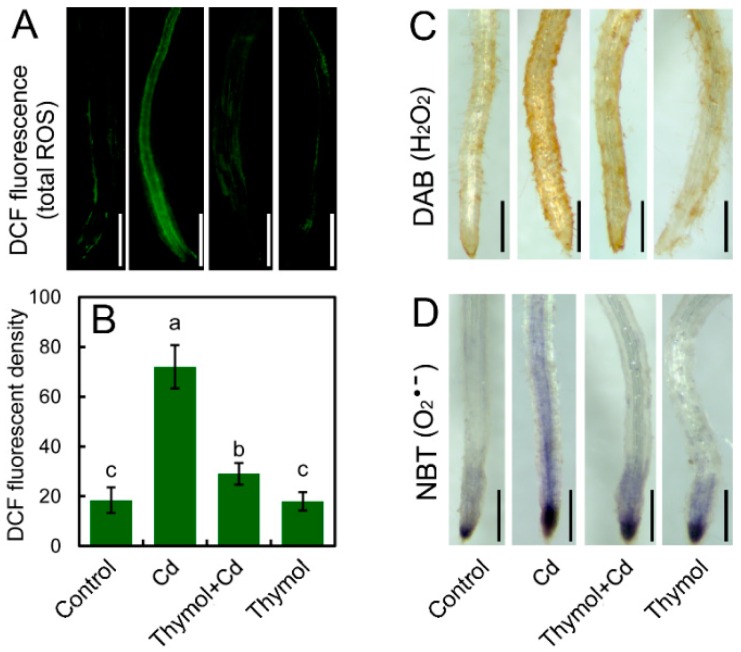
The effect of thymol on ROS generation in the roots of tobacco seedlings under Cd stress. The roots of seedlings were treated with water (control), CdCl_2_ (20 μM), CdCl_2_ (20 μM) + thymol (100 μM), and thymol (100 μM) for 72 h. (**A**) The roots were loaded with DCFH-DA for the observation of total ROS fluorescence with a fluorescent microscope; (**B**) The DCF fluorescent density was quantified to indicate the relative total ROS level in roots; (**C**) The roots were stained with DAB to indicate H_2_O_2_ content; (**D**) The roots were stained with NBT to indicate O_2_^•−^ content. Bar = 1 mm. Different letters in (**B**) indicated that the mean values of three replicates are significantly different between the treatments (*p* < 0.05, ANOVA, LSD).

**Figure 3:**
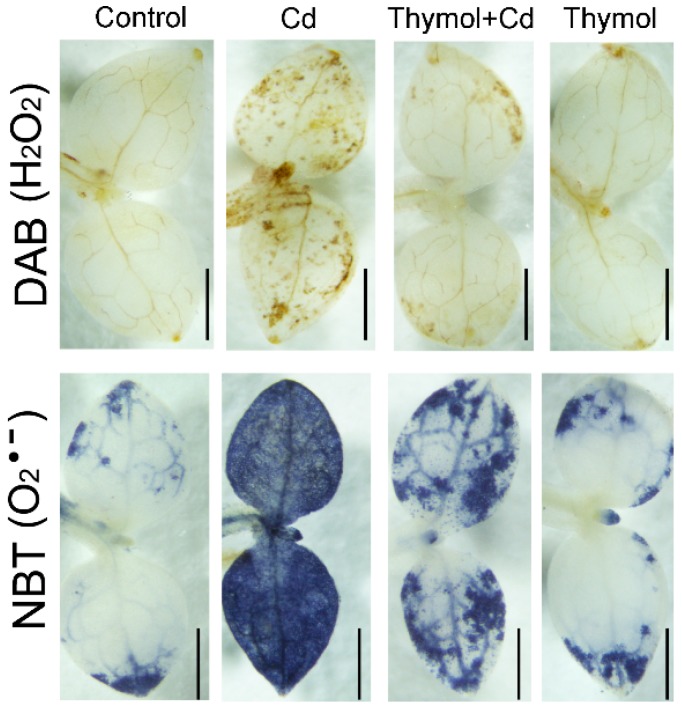
The effect of thymol on the generation H_2_O_2_ and O_2_^•−^ in the leaves of tobacco seedlings under Cd stress. The roots of seedlings were treated with water (control), CdCl_2_ (20 μM), CdCl_2_ (20 μM) + thymol (100 μM), and thymol (100 μM) for 72 h. After treatment, the seedlings were excised at the base of the stem and supplied DAB or NBT for 6 h through the cut stems. Then the leaves were decolorized with ethanol and photographed with a stereoscopic microscope. Bar = 1 mm.

To understand the effect of thymol on ROS generation in roots under Cd stress, total ROS in roots was detected in situ with a specific fluorescent probe, 2′,7′-dichlorofluorescein diacetate (DCFH-DA). Compared to the control, Cd treatment resulted in stronger fluorescence in roots. However, thymol + Cd treatment weakened the fluorescence of total ROS in roots (Figure 2A). The quantification of ROS fluorescent density suggested that the relative total ROS content in Cd-treated roots significantly increased as compared to the control (Figure 2B). Nevertheless, thymol + Cd treatment resulted in the remarkable decrease in the relative total ROS content in roots by 59.63% as compared to Cd treatment alone (Figure 2B). Thymol treatment alone did not significantly affect the content of total ROS in roots (Figure 2B). H_2_O_2_ (hydrogen peroxide) and O_2_^•−^ (superoxide radical) are two typical ROS in plants in response to environmental stimuli [22]. Endogenous H_2_O_2_ in roots was histochemically detected with DAB, which presented as brown. As expected, the generation of H_2_O_2_ in roots increased considerably in response to Cd treatment. However, the addition of thymol inhibited H_2_O_2_ generation in Cd-treated roots (Figure 2C). Particularly, the H_2_O_2_ was decreased in the upper part of the root tip belonging to the differentiation zone (Figure 2C). Endogenous O_2_^•−^ in roots was stained with NBT to present as dark blue. O_2_^•−^ was detected to be located in the root tip, covering the elongation zone (Figure 2D). Thymol decreased the content of O_2_^•−^ in Cd-treated roots (Figure 2D). Cd-induced over-generation of H_2_O_2_ and O_2_^•−^ in leaves were attenuated by thymol, as well (Figure 3). These results suggested that thymol was able to alleviate ROS accumulation in tobacco seedlings under Cd stress.

### 2.3. Thymol Ameliorate Cd-Induced Oxidative Injury in Tobacco Seedlings

**Figure 4:**
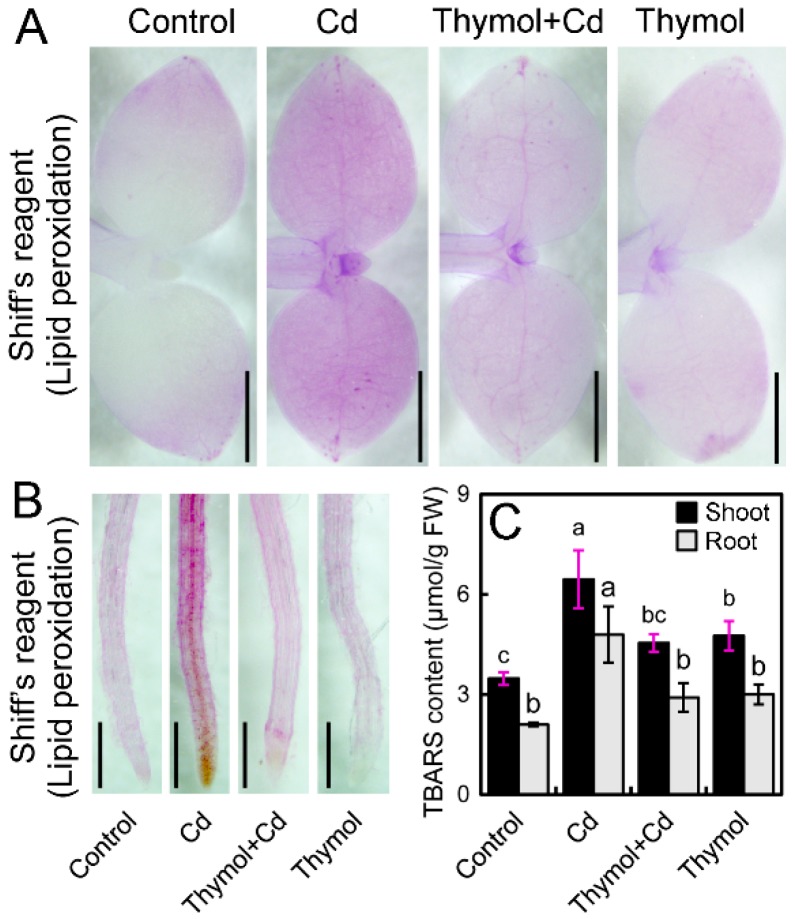
The effect of thymol on lipid peroxidation TBARS content in tobacco seedlings under Cd stress. The roots of seedlings were treated with water (control), CdCl_2_ (20 μM), CdCl_2_ (20 μM) + thymol (100 μM), and thymol (100 μM) for 72 h. (**A**) The seedlings were excised at the base of the stem and supplied Shiff’s reagent or Evans blue for 6 h through the cut stems. Then the leaves were decolorized with ethanol and photographed with a stereoscopic microscope. Bar = 1 mm; (**B**) The roots were stained directly with Shiff’s reagent and photographed with a stereoscopic microscope. Bar = 1 mm; (**C**) Shoots and roots were harvested, respectively, for the measurement of TBARS content. Different letters indicated that the mean values of three replicates are significantly different between the treatments (*p* < 0.05, ANOVA, LSD).

The excessive ROS frequently attacks lipids to induce cell membrane damage that can reflect the level of oxidative injury in plants [22]. In the present study, peroxidation of membrane lipids and the loss of plasma membrane integrity were tested in vivo using histochemical staining with Shiff’s reagent. Cd-treatment alone resulted in extensive staining in leaves, while the leaves treated with thymol + Cd and control had only light staining (Figure 4A). The roots also exhibited similar patterns of lipid peroxidation when compared to leaves upon different treatments (Figure 4B). Thiobarbituric acid reactive substances (TBARS) is a typical product of lipid peroxidation. Cd treatment induced significant increase in TBARS level in both leaves and roots. However, thymol + Cd treatment resulted in the significant decrease in TBARS content by 29.54% and 39.33% in leaves and roots, respectively, as compared to Cd treatment alone (Figure 4C). These results indicated that thymol attenuated Cd-induced oxidative injury in *N. tabacum* seedlings.

### 2.4. Thymol Alleviated Cd-Induced Cell Death in Tobacco Seedlings

**Figure 5:**
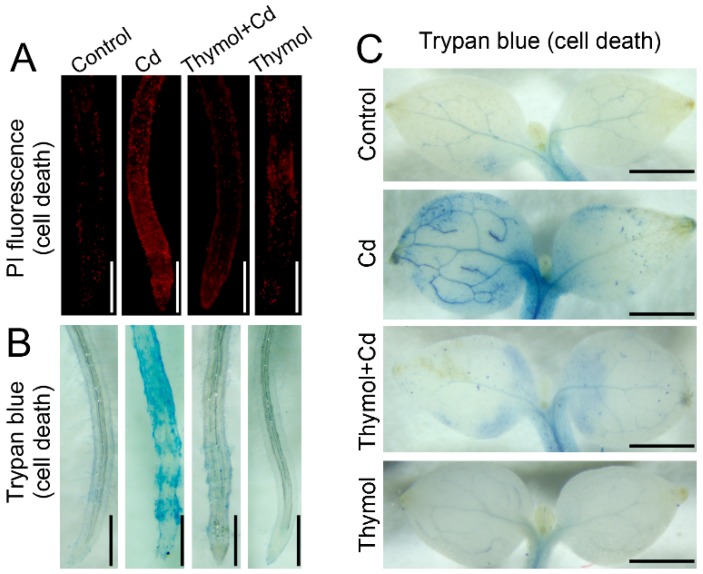
The effect of thymol on cell death in tobacco seedlings under Cd stress. The roots of seedlings were treated with water (control), CdCl_2_ (20 μM), CdCl_2_ (20 μM) + thymol (100 μM), and thymol (100 μM) for 72 h. (**A**) The roots were loaded with PI and photographed with a fluorescent microscope; (**B**) The roots were stained with trypan blue and photographed with a stereoscopic microscope; (**C**) The seedlings were excised at the base of the stem and supplied trypan blue for 6 h through the cut stems. Then the leaves were decolorized with ethanol and photographed with a stereoscopic microscope. Bar = 1 mm.

PI (propidium iodide) staining was performed to indicate cell death in the roots of tobacco seedlings. The PI-stained roots showed more extensive red fluorescence in the presence of Cd than that of the control, while the addition of thymol resulted in lighter fluorescence in Cd-treated roots (Figure 5A). Trypan blue was used as another indicator of cell death. Cd-treatment alone resulted in extensive staining of trypan blue in both roots and leaves as compared to the treatment of thymol + Cd (Figure 5B,C). These results suggested that the addition of thymol led to decreased cell death in tobacco seedlings under Cd stress.

### 2.5. Thymol Decreased Free Cd^2+^ in the Roots of N. tabacum Seedlings

**Figure 6:**
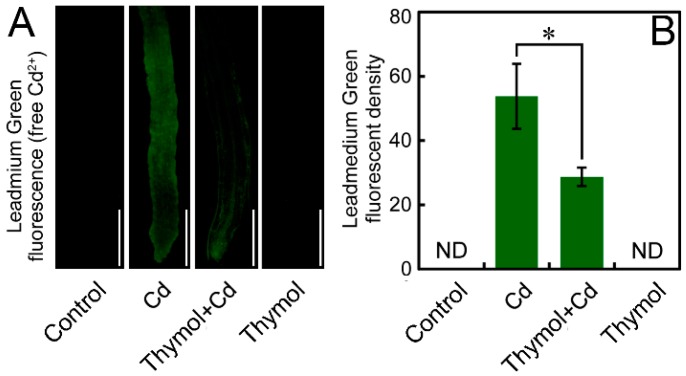
The effect of thymol on free Cd^2+^ in the roots of tobacco seedlings under Cd stress. The roots of seedlings were treated with water (control), CdCl_2_ (20 μM), CdCl_2_ (20 μM) + thymol (100 μM), and thymol (100 μM) for 72 h. Then the roots were loaded with Leadmium™ Green AM for the observation of green fluorescence (**A**) and the quantification of fluorescent density (**B**). Bar = 1 mm. The asterisk in (**B**) indicates that the mean value of three replicates was significantly different between thymol + Cd treatment and Cd treatment alone (*p* < 0.05).

Leadmium™ Green AM (Invitrogen Molecular Probes, Inc., Eugene, OR, USA), with the ability of reacting with free Cd^2+^ to emit green fluorescence, was used to indicate free Cd^2+^ in the roots. In the present study, we did not detect any fluorescent signal in the roots of the control and thymol treatment alone. The roots treated with Cd showed much stronger fluorescence than that of thymol + Cd treatment (Figure 6A). The fluorescent density of Leadmium Green significantly decreased by 46.59% in roots under thymol + Cd treatment as compared to Cd treatment alone (Figure 6B). These results indicated that the addition of thymol resulted in a significant decrease in free Cd^2+^ in Cd-treated roots.

### 2.6. Thymol Enhanced the Content of GSH in the Roots of Cd-Treated Tobacco Seedlings

**Figure 7:**
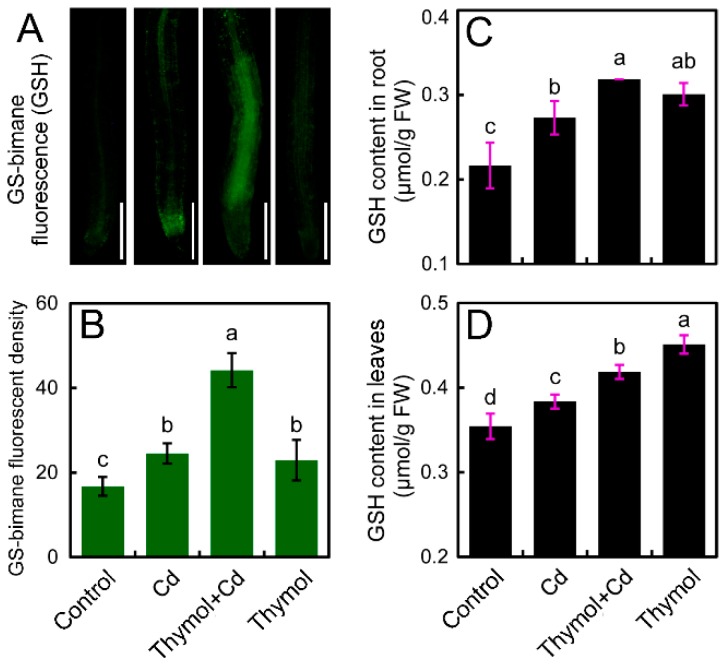
The effect of thymol on GSH content in tobacco seedlings under Cd stress. The roots of seedlings were treated with water (control), CdCl_2_ (20 μM), CdCl_2_ (20 μM) + thymol (100 μM), and thymol (100 μM) for 72 h. Then the roots were loaded with monochlorobimane for the observation of green fluorescence (**A**) and the quantification of fluorescent density (**B**). Bar = 1 mm. The GSH content was determined by in-tube assay in roots (**C**) and leaves (**D**), respectively. Different letters in (**B**–**D**) indicate that the mean values of three replicates are significantly different between the treatments (*p* < 0.05, ANOVA, LSD).

The specific molecular probe monochlorobimane was applied to indicate GSH levels in vivo in the roots of *N. tabacum* seedlings. Cd treatment resulted in the increase in the fluorescence in roots. However, the roots treated with thymol + Cd showed much stronger fluorescence that that of Cd treatment alone (Figure 7A). The strengthened GSH fluorescence was mainly located in the upper part of the root tip (Figure 7A). The fluorescent density in roots under thymol + Cd treatment significantly increased by 80.45% compared to Cd treatment alone (Figure 7B). The in-tube assay of GSH content also suggested that the addition of thymol enhanced GSH content in both roots and leaves in the presence of Cd (Figure 7C,D).

### 2.7. Thymol Stimulated the Expressin of GSH1 and PCS1 in the Roots of Cd-Treated Tobacco Seedlings

**Figure 8:**
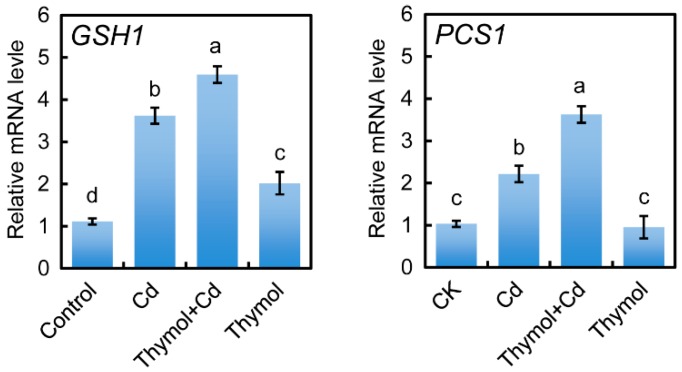
The effect of thymol on the expression of *GSH1* and *PCS1* in the roots of tobacco seedlings under Cd stress. The roots of seedlings were treated with water (control), CdCl_2_ (20 μM), CdCl_2_ (20 μM) + thymol (100 μM), and thymol (100 μM) for 72 h. Then the roots were harvested for RNA extraction and real-time PCR analysis. Different letters indicate that the mean values of three replicates are significantly different between the treatments (*p* < 0.05, ANOVA, LSD).

Real-time reverse transcription-polymerase chain reaction (RT-PCR) was selected to detect the expression of *GSH1* and *PCS1* in the roots of tobacco seedlings upon different treatments. Cd treatment stimulated the expression of *GSH1* and *PCS1* in roots. The addition of thymol still induced the upregulation of the expression of *GSH1* and *PCS1* in Cd-treated roots (Figure 8).

## 3. Discussion

Thymol’s medicinal properties have been drawing great attention because of its physiological regulation on microbial and mammalian cells [23,24]. Essential oils can be developed as green pesticides due to their antimicrobial activity [25], but the regulation of plant physiology by thymol needs to be revealed. It has been reported that thymol is able to protect mammalian cells from metal toxicity [26,27]. Here we demonstrate that thymol confers Cd tolerance in tobacco plants by inhibiting ROS over-generation and decreasing Cd^2+^ accumulation, which is probably dependent on the stimulation of GSH biosynthesis.

ROS accumulation is an important factor contributing to cytotoxicity in plants under Cd stress [28,29,30]. Stress-induced ROS frequently triggers cell death in plants [31]. In the present study, thymol effectively prevented Cd-induced over-generation of ROS (including H_2_O_2_ and O_2_^•−^) in tobacco seedlings, leading to the decrease of cell death and the alleviation of growth inhibition. ROS-induced lipid peroxidation not only directly affects cell membranes, but also aggravates oxidative injury by producing lipid-derived radicals [32]. TBARS is a frequently-used indicator for lipid peroxidation [33]. Here we found that thymol treatment resulted in the significant decrease in both ROS and TBARS content in tobacco seedlings under Cd stress, which may explain the amelioration of Cd-induced lipid peroxidation and oxidative injury by thymol. Thymol has been demonstrated as a ROS quencher by reacting with ROS directly [34,35], but our current results suggested that thymol-stimulated GSH biosynthesis may also contribute to scavenging Cd-induced ROS in tobacco seedlings. GSH plays a critical role in protecting plant cells from oxidative injury because it is located ubiquitously in all organelles, like the endoplasmic reticulum, chloroplasts, cytosol, mitochondria, peroxisomes, vacuole, and apoplast [22]. It has been reported that thymol protects rat erythrocytes and hepatocarcinoma cells from chromium (VI)- and mercury-induced oxidative stress, respectively [26,27]. In these studies, thymol-induced inhibition of ROS accumulation has been closely linked to the enhancement of GSH content in mammalian cells [26,27]. Combined with our current results, it can be speculated that GSH is an important part of the antioxidant properties of thymol against metal toxicity in both mammals and plants. The regulation of GSH by thymol is barely known, however. Here, in Cd-treated tobacco, thymol-induced upregulation of the expression of *GSH1* (a GSH-biosynthetic gene) may partially contribute to the increase in GSH content. GSH content can also be modulated by other physiological metabolism and enzymes, such as the GSH-ascorbate cycle, glutathione reductase (GR), glutathione-*S*-transferase (GST), etc. [22]. The detailed mechanism for the regulation of GSH biosynthesis by thymol needs to be elucidated further.

In addition to the central role of GSH in scavenging ROS, another pivotal function of GSH in the detoxification of heavy metals is to facilitate the chelation of toxic metals in plant cells [36]. GSH-dependent PC biosynthesis is an important strategy developed by plants to combat metal toxicity. PCs can chelate toxic Cd^2+^, followed by compartmentalization in vacuoles, to reduce the aggressive behavior of free Cd^2+^ in other organelles [37]. It has been reported that the transgenic plants with decreased GSH content are highly sensitive to even low levels of Cd^2+^ exposure because of limited PC biosynthesis [38]. In the present study, thymol treatment induced a remarkable decrease in free Cd^2+^ detected with a specific fluorescent probe in vivo, coinciding with the increase in GSH content and the upregulation of *PCS1* (a PCs-biosynthetic gene) in the roots of tobacco seedlings upon Cd exposure. In Cd-treated roots, thymol seems to activate GSH-PCs pathway in two ways. First, thymol stimulated the biosynthesis of GSH, providing more precursors for the biosynthesis of PCs. Second, thymol directly activated the biosynthesis of PCs by upregulating the expression of *PCS1*. In addition, the results from fluorescent microscope suggested that the increased GSH accumulation shared the same region with the decreased Cd^2+^ in the roots. Thus, it can be speculated that the thymol-activated GSH-PCs pathway arrests free Cd^2+^ in roots, which may further limit the transportation of Cd to shoots to alleviate Cd-induced toxicity in leaves. These findings provide a novel clue for identifying the action of thymol on metal detoxification in plants. The detailed mechanism for the regulation of the GSH-PCs pathway by thymol would be an interesting topic for further study.

Mitochondria and apoplasts are two major locations of ROS production in plants under both biotic and abiotic stresses [22,39]. Stress-conditions always result in the dysfunction of mitochondria by inducing the disturbance of the mitochondrial electronic transfer chain (ETC), leading to the direct oxygen reduction for ROS production [40]. The rapid ROS generation has been found in isolated mitochondria from potato tubers under Cd stress [41]. In apoplasts, NADPH (Nicotinamide Adenine Dinucleotide Phosphate) oxidase located in the plasma membrane has been demonstrated to be a major source for ROS generation [42]. In has been reported that Cd-induced tobacco cell death is attributed to NADPH oxidase-dependent ROS generation [28,43]. In mammals, thymol is capable of abolishing mitochondrial dysfunction in isoproterenol-induced myocardial infarcted rats by inhibiting oxidative stress [16]. In addition, thymol-inhibited ROS generation and NADPH oxidase activity have been found in lipopolysaccharide-stimulated macrophages [44]. In Cd-treated tobacco seedlings, whether thymol inhibits ROS generation through the similar mechanisms needs to be investigated.

In the present study, the addition of thymol stimulated the growth of Cd-treated seedlings, but thymol treatment alone showed a slight inhibitory effect on seedling growth as compared to the control. It has been reported that thymol can inhibit seedling growth of several plant species [45,46]. Thymol-induced growth inhibition of maize seedlings has been linked to the occurrence of lipid peroxidation resulting from the alteration of fatty acid composition [46]. Here we also found that thymol treatment led to the increase in TBARS content in tobacco seedlings under normal growth conditions. However, the addition of thymol was able to remarkably decrease Cd-induced TBARS in tobacco seedlings. The possible explanation is that thymol has a strong capability to scavenge Cd-induced ROS, leading to the alleviation of subsequent lipid peroxidation, and this process may overcome the inducible effect of thymol itself on lipid peroxidation. Nevertheless, further studies are needed to compare and distinguish the protective effect of thymol under stress conditions and the possible inhibitory effect of thymol under normal conditions.

## 4. Materials and Methods

### 4.1. Plant Culture and Treatment

Seeds of *N. tabacum* were germinated for one day in the dark on the wet filter paper in a Petri dish. Then the selected identical seedlings with radicles 0.5 cm were transferred to another Petri dish containing various treatment solutions in a chamber with a photosynthetic active radiation of 200 μmol/m^2^/s, a photoperiod of 12 h, and the temperature at 25 °C. According to different experimental designs, the roots of the seedling were exposed to water, CdCl_2_ (5–80 μM), thymol (10–400 μM), alone, or combined solutions for various treatment times (0–72 h). Then the shoots and roots were harvested, respectively, and washed with distilled water for histochemical, physiological, and biochemical analysis.

### 4.2. Histochemical Analysis of Total ROS in Roots

Intracellular ROS in roots was visualized using a specific fluorescent probe, DCFH-DA in situ, as described by Foreman et al. [47]. The roots of seedlings were incubated in 10 μM of DCFH-DA at 25 °C for 10 min. Then the roots were rinsed with distilled water three times, followed by visualization (excitation 488 nm and emission 525 nm) with a fluorescence microscope (ECLIPSE, TE2000-S, Nikon, Melville, NY, USA). The relative fluorescent density of the fluorescent images was analyzed using Image-Pro Plus 6.0 (Media Cybernetics, Inc., Rockville, MD, USA).

### 4.3. Histochemical Detection of Intracellular H_2_O_2_ in Roots and Leaves

Histochemical detection of endogenous H_2_O_2_ in roots was performed by using 3,3-diaminobenzidine (DAB) staining, as described by Nguyen et al. [48]. The roots of seedlings after treatment were transferred to 0.1% (*w*/*v*) of DAB–HCl solution (pH 3.8) for incubation for 20 min. Then the roots were rinsed with distilled water three times, which allowed a deep brown polymerization product (reaction of DAB and H_2_O_2_) to be clearly visualized and photographed under a stereoscopic microscope (SteREO Discovery.V8, ZEISS, Oberkochen, Germany). For the histochemical detection of H_2_O_2_ in leaves, the treated seedlings were excised at the base of the stems with a razor blade and supplied 0.1% (*w*/*v*) of DAB–HCl solution (pH 3.8) for 6 h through the cut stems [49]. Then the leaves were transferred to boiling ethanol for 20 min to remove the green background. After that, the leaves were observed and photographed with a stereoscopic microscope (SteREO Discovery.V8, ZEISS).

### 4.4. Histochemical Detection of Intracellular O_2_^•−^ in Roots and Leaves

Histochemical detection of endogenous O_2_^•−^ in roots was performed by using nitro-blue tetrazolium (NBT) staining, as described by Frahry and Schopfer [50]. The roots of seedlings after treatment were transferred to 10 mM Na-citrate buffer (pH 6.0) containing 6 mM NBT under light at 25 °C for 20 min, and then the roots were rinsed with distilled water three times, which allowed the dark blue insoluble formazan compound (by reaction of NBT with O_2_^•−^) inside the roots to be clearly visualized and photographed under a stereoscopic microscope (SteREO Discovery.V8, ZEISS). For the histochemical detection of O_2_^•−^ in leaves, the treated seedlings were excised at the base of the stems with a razor blade and supplied 6 mM NBT solution for 6 h through the cut stems [49]. Then the leaves were transferred to boiling ethanol for 20 min to remove the green background. After that, the leaves were observed and photographed with a stereoscopic microscope (SteREO Discovery.V8, ZEISS).

### 4.5. Histochemical Detection of Lipid Peroxidation in Roots and Leaves

Histochemical detection of lipid peroxidation was achieved by using Schiff’s regent, as described by Wang and Yang [51]. The roots of seedlings after treatment were incubated in Schiff’s reagent for 20 min. Then the stained roots were rinsed with a solution containing 0.5% (*w*/*v*) K_2_S_2_O_5_ (prepared in 0.05 M of HCl) until the root color became light red. After that, the roots were photographed by using a stereoscopic microscope (SteREO Discovery.V8, ZEISS). For the histochemical detection of lipid peroxidation in leaves, the treated seedlings were excised at the base of the stems with a razor blade and supplied Schiff’s reagent for 6 h through the cut stems. Then the leaves were incubated in K_2_S_2_O_5_ solution followed by transferring to boiling ethanol for 20 min to remove the green background. After that, the leaves were observed and photographed with stereoscopic microscope (SteREO Discovery.V8, ZEISS).

### 4.6. Determination of TBARS Content

The concentration of TBARS was determined as an indicator of the level of lipid peroxidation in plants. A TBRAS detection kit (A003; Nanjing Jiancheng Bioengineering Institute, Nanjing, China) was selected to measure the TBARS level based on the spectrophotometric determination of the reaction between TBARS and 1,3-diethyl-2-thiobarbituric acid (TBA) assisted by trichloroacetic acid (TCA) [52].

### 4.7. Histochemical Detection of Cell Death in Roots and Leaves

Histochemical detection of cell death in roots was performed by using the fluorescent probe propidium iodide (PI) in situ, as described by Kellermeier et al. [53]. The roots of seedlings after treatment were incubated in 20 μM of PI solution for 20 min. Then the roots were rinsed with distilled water three times and were visualized (excitation 535 nm and emission 615 nm) by a fluorescence microscope (ECLIPSE, TE2000-S, Nikon).

Cell death was also confirmed by histochemical staining with trypan blue [54]. The roots of seedlings after treatment were incubated in 10 mg/mL of trypan blue solution for 20 min. After that, the roots were rinsed with distilled water three times followed by imaging with a stereoscopic microscope (SteREO Discovery.V8, ZEISS). For the detection of cell death in leaves, the treated seedlings were excised at the base of stems with a razor blade and were supplied with trypan blue for 6 h through the cut stems. Then the leaves were incubated in boiling ethanol for 20 min to remove the green background followed by photographing with a stereoscopic microscope (SteREO Discovery.V8, ZEISS).

### 4.8. Histochemical Detection of Free Cd^2+^ in Roots

Free Cd^2+^ in roots was detected in vivo by using fluorescent probe Leadmium™ Green AM [55]. The roots of seedlings were incubated in 1 μg/mL of Leadmium™ Green AM at 25 °C for 20 min. Then the roots were rinsed with distilled water three times, followed by visualization (excitation 488 nm and emission 525 nm) with a fluorescence microscope (ECLIPSE, TE2000-S, Nikon).

### 4.9. Histochemical Detection of GSH in Roots

Histochemical detection of glutathione (GSH) was performed by using the specific molecular probe monochlorobimane in situ, as described by Liso et al. [56]. The endogenous GSH in roots was visualized after conjugation with monochlorobimane to give fluorescent GS-bimane adducts. The roots of the seedlings after treatment were incubated in 100 μM of monochlorobimane solution for 30 min. Then the roots were rinsed with distilled water three times and were visualized (excitation 390 nm and emission 478 nm) by a fluorescent microscope (ECLIPSE, TE2000-S, Nikon). The relative fluorescent density of the fluorescent images was analyzed using Image-Pro Plus 6.0 (Media Cybernetics, Inc.).

### 4.10. Determination of GSH Content in Roots and Leaves

GSH content in the samples of roots or shoots was determined by using a GSH assay kit (A006-1; Nanjing Jiancheng Bioengineering Institute, Nanjing, China) according to the manufacturer’s instructions [57]. GSH content was determined based on the spectrophotometric determination of the reaction between GSH and 5,5′-dithiobis(2-nitrobenzoic acid) (DTNB) for 420 nm.

### 4.11. Analysis of Gene Expression

Real-time RT-PCR was selected to quantify the expression levels of the genes. The sequences of *GSH1* (accession number SGN-U423511) and *PCS1* (accession number SGN-U48649) were retrieved from Sol Genomics Network (https://solgenomics.net/) for the design of primers. Total RNA was extracted from roots using Trizol (Invitrogen) according to the manufacturer’s instructions. Reverse transcription was performed at 42 °C in 25 μL reaction mixture including 3 μg of RNA, 0.5 μg of oligo (dT) primers, 12.5 nmol of dNTPs, 20 units of RANase inhibitor and 200 units of M-MLV. The first cDNA was used as a template for real-time RT-PCR analysis (Applied Biosystems 7500 Fast Real-Time PCR System, LifeTechnologies™). The primers used for amplifying the target genes are as follows: *GSH1*, forward 5′-GAGGATAGGCACTGAACATGAA-3′ and reverse 5′-TCGCTCGGCAATACCATTTAG-3′; *PCS1*, forward 5′-GCTGGGTGGGTTCAGATTTA-3′ and reverse 5′-TTCCTTCAGCTCTTGTCAGAAT-3′. To standardize the results, the relative abundance of *EF1-α* (*Elongation Factor 1-α*, forward 5′-ATGATGACGACGATGATGATA-3′ and reverse 5′-GTAAGCCCTTCTTGCTGAACAC-3′) was also determined and used as the internal standard.

### 4.12. Data Analysis

Each result was presented as the mean ± standard deviation (SD) of at least three replicated measurements. The significant differences between treatments were statistically evaluated by SD and one-way analysis of variance (ANOVA) using SPSS 14.0 (Statistical Package for the Social Science, SPSS Inc., Chicago, IL, USA). The data between two specific different treatments were compared statistically by ANOVA, followed by an *F*-test if the ANOVA result is significant at *p* < 0.05. For multiple comparison analysis, a least significant difference test (LSD) was performed on all data following ANOVA tests to test for significant (*p* < 0.05) differences among different treatments.

## 5. Conclusions

In sum, we demonstrated that thymol was able to protect tobacco seedlings from Cd stress. Thymol-induced GSH biosynthesis contributed to the reestablishment of ROS homeostasis and the abrogation of free Cd^2+^ accumulation, oxidative injury, and cell death in tobacco seedlings. The detailed mechanism for thymol-facilitated plant adaption to Cd is largely unknown, but the current results provide evidence for the regulation of plant-resistant physiology by thymol. This would extend our knowledge for the possible application of thymol in agriculture by helping plants combat environmental stress. However, thymol possesses strong activity against microbes including some plant pathogens and soil microflora [13,58,59]. In the agricultural environment, the possible influence of thymol on the microbial community should be a regulatory concern. Moreover, the feasibility of applying thymol in agriculture needs to be verified by more field tests for different plant species in different agricultural environments.
